# Clinical efficacy of intrauterine platelet-rich plasma infusion in endometrial regeneration after hysteroscopic adhesiolysis: A retrospective cohort study

**DOI:** 10.1097/MD.0000000000043754

**Published:** 2025-08-08

**Authors:** Hui-Liu Fan, Xiao-Xia Wu, Hai-Chun Wei, Ying Zhang, Miao-Ling Dou, Hong-Lan Wei

**Affiliations:** a Department of Gynaecology, Liuzhou Maternity and Child Healthcare Hospital, Guangxi, China; b Department of Gynaecology, Guangzhou Women and Children’s Medical Center Liuzhou Hospital, Guangxi, China.

**Keywords:** platelet-rich plasma, intrauterine infusion, intrauterine adhesion, thin endometrium

## Abstract

The study investigated the effects of intrauterine infusion of platelet-rich plasma (PRP) on endometrial growth, repair of thin endometrium, and pregnancy outcomes following surgery for intrauterine adhesions (IUA). Fifty patients with severe IUA underwent hysteroscopic transcervical resection of adhesions (TCRA). Patients with normal uterine cavity morphology but thin endometrium on the second examination were selected for this observational study and divided into 2 groups of 25 each. The experimental group received intrauterine infusion of autologous PRP to promote endometrial growth, along with hormone replacement therapy, while the control group received only artificial menstrual cycle treatment. Endometrial thickness and blood flow values were observed in both groups 3 months post-surgery, and pregnancy outcomes were recorded within 1-year post-surgery. The experimental group showed slightly greater endometrial thickness than the control group; however, *P* > .05 indicated no statistically significant difference. Similarly, there was no significant difference in resistance index values between the 2 groups (*P* > .05). The pregnancy rate in the experimental group (36%) was higher than that in the control group (20%), but with *P* > .05, indicating no statistically significant difference. Intrauterine infusion of PRP for treating thin endometrium following severe IUA surgery did not significantly increase endometrial thickness or improve pregnancy rates.

## 1. Introduction

IUA, also known as Asherman syndrome (AS), is caused by infection, intrauterine surgery, and other factors that damage the basal layer of the endometrium, leading to adhesion formation. Clinical manifestations include hypomenorrhea, amenorrhea, infertility, recurrent miscarriage, cyclical abdominal pain, and placenta accreta.^[1]^ According to guidelines from the American Association of Gynecological Laparoscopy and the European Society of Gynecological Endoscopy, treatment primarily involves surgical intervention and postoperative prevention of adhesion recurrence, which includes the use of solid and semisolid barriers and hormone therapy.^[2]^ However, studies have indicated that hormone therapy, intrauterine balloons, intrauterine devices, hyaluronic acid gel, and amniotic membrane provide limited therapeutic benefits in preventing IUA recurrence and do not significantly improve clinical symptoms or pregnancy rates.^[3–5]^ There remains a risk of recurrence, repeated miscarriage, and failed embryo implantation after surgery.^[6–8]^ Research has shown that the rate of adhesion recurrence following severe IUA surgery can reach up to 62.5%,^[9]^ often resulting in pregnancy-related complications and a low live birth rate. Therefore, adjuvant therapy after IUA surgery, particularly after severe cases, has focused on promoting endometrial growth, reducing adhesion recurrence, and enhancing reproductive outcomes.

Currently, treatment methods for thin endometrium include hormone replacement therapy, oral medications to improve endometrial blood flow (e.g., aspirin, vitamin E, sildenafil citrate, pentoxifylline), hysteroscopy, pelvic floor neuromuscular electrical stimulation, growth hormone therapy, and stem cell therapy; however, these approaches have shown limited effectiveness.^[10,11]^ Thus, finding effective treatments for thin endometrium is of considerable clinical importance. The rapid development of regenerative medicine has introduced new strategies for treating thin endometrium, particularly patient-derived cell-based therapies.

PRP is a type of plasma rich in platelets, obtained by centrifuging whole blood to achieve a high concentration of platelets, several times that of whole blood. PRP contains numerous growth factors (GFs) and proteins that facilitate tissue repair, regeneration, angiogenesis, immune regulation, and anti-inflammatory effects, among other biological functions.^[12]^ Autologous PRP, with its high concentration of GFs and cytokines, stimulates mitosis and proliferation of endometrial cells or endometrial stem cells, thereby activating the paracrine pathway, enhancing endometrial response, and supporting embryo implantation and pregnancy.^[13]^ PRP also reduces fibrosis from endometrial injury, increases the local anti-inflammatory response in the uterine cavity, and improves endometrial receptivity.^[14]^ Injecting PRP at the injury site releases bioactive factors (e.g., GFs, cytokines, lysosomes) and adhesion proteins that initiate the hemostasis cascade, regulate the biological microenvironment of the injured site, and trigger tissue regeneration, connective tissue synthesis, and blood circulation reconstruction.^[15]^ Additionally, PRP is easy to prepare, safe, reliable, and ethically acceptable, and has been widely applied in medical fields like hair regeneration and wound healing.^[16]^ These advantages and applications suggest that PRP may be beneficial in treating thin endometrium.

Current research shows that intrauterine infusion drug therapy can increase endometrial thickness and improve receptivity to some extent. Compared to intravenous administration, intrauterine infusion delivers a high concentration of the drug directly to the endometrium, reducing loss through peripheral blood circulation. This approach enhances endometrial receptivity, promotes increased endometrial thickness, regulates the uterine cavity’s internal immune microenvironment, and raises embryo implantation rates. Some studies have examined intrauterine infusion of colony-stimulating factors, peripheral blood mononuclear cells, human chorionic gonadotropin, and stem cell therapies in patients with thin endometrium, showing improved pregnancy outcomes; however, some researchers noted that intrauterine drug infusion did not increase endometrial thickness.^[11]^ Theoretically, PRP, containing cytokines, various proteins, and GFs, supports cell proliferation, neovascularization, and anti-inflammatory effects. Applying PRP to the endometrium can enhance endometrial receptivity, increase thickness, and thereby improve pregnancy rates and outcomes.^[17]^

Intrauterine infusion of PRP is a novel approach recently explored for treating thin endometrium. However, its therapeutic effect on thin endometrium resulting from trauma and adhesion scars remains unclear. This study aimed to evaluate PRP intrauterine infusion as a treatment for patients with thin endometrium post-adhesion surgery. By observing changes in endometrial thickness, uterine artery blood flow parameters, and pregnancy outcomes, this study sought to assess the therapeutic effect of PRP intrauterine infusion on patients with thin endometrium following adhesion surgery. The goal was to provide a safe and effective clinical treatment plan for such patients.

## 2. Materials and methods

### 2.1. Patients

This study retrospectively analyzed 50 patients with severe IUA who underwent hysteroscopic adhesiolysis at Liuzhou Maternity and Child Healthcare from July 2021 to June 2023. Inclusion criteria included: confirmation of normal uterine cavity morphology on second-look hysteroscopy, but with pale or thin endometrium; endometrial thickness ≤7 mm on ovulation day in at least 2 consecutive natural cycles postoperatively; age ≤40 years with active fertility intentions. Participants were allocated into experimental (n = 25) and control (n = 25) groups based on their consent or refusal of the proposed adjuvant therapy. This study was reviewed and approved by the hospital’s ethics committee (No. Exp-SR-2021-048), and all participants provided informed consent with signed treatment agreements.

Contraindications included: contraindications to estrogen and progesterone use, such as thrombotic diseases, estrogen-dependent tumors or diseases, unexplained abnormal uterine bleeding, liver and kidney dysfunction, diabetes, depression; presence of uterine fibroids, adenomyosis, or uterine deformity; acute inflammation or systemic diseases intolerant to surgery; smoking (≥10 cigarettes/day); mental conditions impairing treatment compliance; incomplete treatment or loss to follow-up; presence of endometrial tuberculosis.

Comparisons of age, coexisting infertility, menstrual changes, and obstetric history between the 2 groups revealed no statistically significant differences (*P* > .05) (see Table 1).

**Table 1:** Comparison of general information between the 2 groups ($\bar{x}\pm \;s$).

Group	Number of cases (n)	Number of infertility Cases (n)	Age (year)	Menstrual change duration (months)	Pregnancy and delivery history
Experimental	25	15	30.5 ± 4.5	14.30 ± 7.72	2.57 ± 1.06
Control	25	13	29.4 ± 6.7	13.43 ± 9.7	2.53 ± 1.09
*P*	–	>.05	>.05	>.05	>.05

#### Experimental group

Hysteroscopic infusion of PRP was performed once during the 3 to 7 days following menstruation, and again 1 week later. The treatment was repeated for an additional cycle in the following month, alongside 2 cycles of artificial menstrual cycle treatment.

#### Control group

Two cycles of artificial menstrual cycle treatment were administered.

### 2.2. HRT

Estradiol Valerate tablets (1 mg/tablet, Bayer HealthCare Pharmaceuticals, Germany) were administered at 2 mg twice daily for 21 days, supplemented with Dydrogesterone tablets (10 mg/tablet, Abbott Healthcare (Shanghai) Trading Co., Ltd.) at 10 mg once daily during the final 10 days of the cycle. This regimen was repeated every 21 days to induce cyclic withdrawal bleeding.

### 2.3. Preparation of PRP

Peripheral blood (10 mL) was drawn from the patient with a 10 mL syringe and sent to the laboratory. After adding an anticoagulant, the sample was centrifuged at a force of 2000 × g for 10 minutes, resulting in 3 layers: the upper layer (plasma), the middle layer (platelets and white blood cells), and the bottom layer (red blood cells). The red blood cell layer and plasma were removed, leaving the PRP. This PRP was then mixed with calcium gluconate and thrombin in an appropriate ratio to form a platelet-rich gel, which induced the release of bioactive molecules from α-granules.^[18]^

One mL of prepared PRP gel was drawn and connected to an artificial insemination catheter. Under abdominal B-mode ultrasound monitoring, the gel was slowly injected into the uterine cavity, left to stay for 15 seconds, and then the catheter was removed.

### 2.4. Observation

During the treatment period and at 1, 2, and 3 months, as well as 1-year posttreatment, follow-ups were conducted. Follow-up assessments included measuring endometrial thickness and endometrial blood flow during the ovulatory cycle using transvaginal color Doppler ultrasound and tracking pregnancy outcomes within 1 year.

### 2.5. Statistical methods

Data were analyzed using SPSS 19.0 statistical software (Chicago). Quantitative data were expressed as mean ± standard deviation (x ± s), with group comparisons conducted using the independent *t*-test. Categorical data were expressed as percentages (%), and group comparisons were made using the chi-square test. A *P*-value of <.05 was considered statistically significant.

## 3. Results

Both groups underwent transvaginal color Doppler ultrasound monitoring of endometrial thickness and resistance index during the mid-luteal phase posttreatment. Results indicated that endometrial thickness in the experimental group was slightly greater than in the control group; however, with *P* > .05, the difference was not statistically significant. Comparison of resistance index values between the groups also showed no significant difference (*P* > .05) (see Table 2). The pregnancy rate in the experimental group was 36%, higher than the control group’s 20%, but with *P* > .05, indicating no statistically significant difference (see Table 3).

**Table 2:** Comparison of endometrial thickness and blood flow after treatment between the 2 groups ($\bar{x}\pm \;s$).

Group	Endometrial thickness (cm)			Endometrial blood flow index (RI)		
	1 mo	2 mo	3 mo	1 mo	2 mo	3 mo
Experimental	0.55 ± 0.13	0.61 ± 0.15	0.62 ± 0.12	0.49 ± 0.05	0.47 ± 0.03	0.44 ± 0.01
Control	0.53 ± 0.11	0.55 ± 0.13	0.57 ± 0.14	0.53 ± 0.03	0.51 ± 0.04	0.46 ± 0.04
*P*	>.05	>.05	>.05	>.05	>.05	>.05

RI = resistance index.

**Table 3:** Comparison of pregnancy outcomes between the 2 groups (n[%]).

Group	Number of cases (n)	Natural pregnancy (n)	IVF (n)	Total pregnancy rate (%)
Experimental	25	4	5	36%
Control	25	2	3	20%
*χ* ^*2*^	–	–	–	1.587
*P*	–	–	–	.208

IVF = in vitro fertilization.

Treatment of thin endometrium after severe intrauterine adhesion surgery with intrauterine infusion of platelet-rich plasma (PRP) did not significantly increase endometrial thickness or improve pregnancy rates.

## 4. Discussion

The high recurrence rate of adhesions and low pregnancy rates following IUA surgery are closely related to poor endometrial repair, particularly in patients with thin endometrium after severe IUA. Comprehensive treatment of IUA should not only focus on removing adhesion bands and preventing their reformation but should also prioritize endometrial repair and regeneration. This approach aims to restore endometrial function and create a favorable uterine environment for future pregnancies.

PRP, also referred to as platelet-rich GFs, has a platelet concentration that is 5 to 7 times higher than that of peripheral blood, and was initially prepared as a blood product for treating thrombocytopenia.^[19]^ Research by Amable et al demonstrated that PRP is rich in platelet-derived GFs (PDGFs), epidermal growth factor (EGF), transforming growth factor, and cytokines. During wound healing, activated platelets release these GFs, which synergistically promote wound repair.^[20]^ Since then, PRP has been widely applied in various medical fields, including tissue regeneration, wound healing, ligament repair, and hair loss.^[21]^ In 2015, Chang and colleagues first reported that intrauterine infusion of PRP could promote endometrial growth and improve pregnancy outcomes in patients with thin endometrium, pioneering PRP use for treating gynecological conditions in women.^[22]^ The clinical study conducted by Professor Duan Hua team also demonstrated that postoperative intrauterine infusion of PRP in patients with moderate-to-severe IUA can effectively reduce readhesion formation and improve menstrual volume as well as endometrial thickness.^[14]^ Additionally, it enhances the levels of multiple cytokines in uterine cavity drainage fluid within 48 hours after TCRA. This treatment promotes microvascular formation at intrauterine wound sites and facilitates endometrial regeneration and repair, thereby contributing to reduced postoperative readhesion formation and improved treatment prognosis.^[23]^

The mechanisms by which PRP treats thin endometrium are complex, with scholars proposing several possible pathways^[24]^: PRP promotes cell regeneration, proliferation, and angiogenesis through GFs such as vascular endothelial growth factor, PDGF, transforming growth factor-βI (TGF-βI), EGF, insulin-like growth factor 1 (IGF-1), and hepatocyte growth factor, and it stimulates expression of chemokines like CXC chemokine ligand 5 (CXCL5) and CXCL12; activated PRP promotes migration of human endometrial stem/progenitor cells, such as menstrual blood stem/stromal cells (MenSC), endometrial mesenchymal stem cells (eMSC), and bone marrow-derived mesenchymal stem cells (BMSC); PRP facilitates cellular migration via chemotaxis, promoting mesenchymal-to-epithelial cell transformation; it increases paracrine factor levels and reduces cell apoptosis by activating the PDGF receptor/phosphoinositide 3-kinase (PI3K)/protein kinase B (AKT)/nuclear factor-kB (NF-kB) signaling pathway, thereby enhancing cell regeneration.^[25]^

Current observational studies indicate that intrauterine perfusion of PRP demonstrates multiple advantages in the treatment of IUA post-surgery: high safety: it utilizes autologous peripheral blood, avoiding risks of immune rejection, blood-borne diseases, and complications associated with allogeneic transfusion. Rich in GFs^[12]^: PRP contains abundant GFs that promote endometrial cell proliferation, differentiation, angiogenesis, and antifibrotic effects, thereby improving the local microenvironment and endometrial receptivity while supporting tissue regeneration. Clinical efficacy: PRP enhances endometrial growth, improves endometrial thickness, and reduces recurrence of intrauterine adhesions.^[14,17,23]^ Convenient procedure: the treatment is minimally invasive, requiring no cervical dilation or surgical intervention. PRP is delivered into the uterine cavity via a catheter, with a short procedure time and no need for hospitalization, resulting in good patient compliance. Cost-effectiveness: PRP preparation is low-cost and does not require complex equipment, making it suitable for widespread clinical application. Therefore, PRP is expected to be used as an adjunct treatment after the separation of IUA to promote endometrial repair, reduce adhesion recurrence, and improve reproductive outcomes in infertile patients.

However, in this study on the treatment of thin endometrium post-severe IUA surgery with PRP infusion, endometrial thickness between the 2 patient groups was compared. Although endometrial thickness slightly increased in the experimental group, the difference was not statistically significant (*P* > .05). A 2020 randomized controlled trial with a small sample size similarly found that PRP infusion did not significantly improve menstrual volume or increase endometrial thickness, nor did it reduce postoperative IUA scores relative to the control group.^[26]^ In contrast, in 2015, Liang Xiaoyan team at the 6th Affiliated Hospital of Sun Yat-sen University, China, was the first to report that PRP infusion positively influenced endometrial repair and improved reproductive outcomes in patients with intrauterine adhesion-related infertility.^[22]^ Subsequently, Aghajanova et al^[27]^ reported in a series of case studies that 2 patients with IUA successfully conceived after postoperative PRP infusion, despite no significant increase in endometrial thickness; however, only 2 cases were included in this report.

Regarding pregnancy outcomes, no statistically significant difference was observed in pregnancy rates between the 2 groups in this study (*P* > .05). A review of related research found that a retrospective cohort study by Peng et al^[28]^ compared postoperative IUA scores and pregnancy rates between PRP and balloon groups. The pregnancy rate in the PRP group was higher than in the balloon group, although the difference was not statistically significant. Similarly, a 2022 randomized controlled trial by a research team affiliated with Capital Medical University in China (n = 126) showed that adjunctive PRP infusion after moderate-to-severe IUA surgery significantly reduced adhesion scores. However, PRP did not significantly lower adhesion recurrence or increase pregnancy rates compared to the control group.^[14]^ Another study reported a case of severe IUA and thin endometrium where, following 2 PRP infusions, the patient’s endometrial thickness increased on color Doppler ultrasound, with marked improvement in gland development observed on a second hysteroscopy, ultimately resulting in successful pregnancy and delivery. This suggests that PRP may not only promote endometrial growth but also support embryo implantation^[13]^

On the timing and method of PRP application, several studies have shown that PRP can be administered immediately after IUA release, primarily through intrauterine infusion,^[14,28–30]^ or combined with intramuscular injection at sites of severe endometrial damage,^[31–33]^ using treatment doses ranging from 0.5 to 10 mL per application. Both intrauterine infusion and combined intramuscular injection appear effective in enhancing pregnancy rates, reducing adhesion recurrence, and improving menstrual patterns. Most studies suggest that postoperative PRP infusion may improve endometrial function in patients with IUA, regulate the uterine microenvironment, and increase pregnancy rates. However, the use of PRP in IUA treatment remains exploratory, with limited sample sizes in existing studies.

In this study, intrauterine infusion of PRP did not significantly increase endometrial thickness or improve pregnancy rates. This may be attributed to the following factors: all cases in this study were post-severe IUA surgery, with pale and scant scarred endometrial tissue, severe lesions, basal layer damage, and scar tissue formation, making it challenging to enhance vascularity and regenerative function of the uterine base within a short treatment period. The number of IUA may have been insufficient. In this study, PRP was infused into the uterus 3 to 7 days post-menstruation, and again 1 week later, repeated over 2 cycles. However, the expert consensus on treating thin endometrium with autologous PRP, published in June 2023,^[34]^ suggests that if the target endometrial thickness is not reached during treatment, PRP can be infused every 2 to 3 days, with a typical course comprising 3 to 5 infusions. Thus, single-infusion volume, interval between infusions, and total infusions may influence the microenvironment for endometrial repair. The intrauterine infusion method may require improvement. For patients with refractory thin endometrium, direct intrauterine PRP injection can be considered. Experts recommend using an egg retrieval or puncture needle to inject PRP into the severely damaged lower endometrial layer, with an injection depth of approximately 1 cm and a dose of 0.5 to 1 mL. In 2020, Agarwal et al^[35]^ first suggested that PRP injection into the subendometrial area under hysteroscopic guidance could significantly enhance endometrial thickness and local blood supply. The PRP preparation dose may have been insufficient. In this study, 10 mL of the patient’s blood was drawn to prepare 1 mL of PRP via centrifugation, which may not have provided an adequate concentration. Chinese expert consensus recommends drawing around 40 mL of blood to meet the treatment amount for 1 course.^[34]^ There is no standardized preparation method for PRP, and platelet concentration and bioactive factor levels can vary significantly depending on the preparation technique. These variations may relate to the concentration of white blood cells and the factors they produce. Higher white blood cell concentrations in PRP correlate with increased levels of vascular endothelial growth factor, hepatocyte growth factor, IGF-1, and PDGF-AB,^[36]^ potentially impacting PRP efficacy. In this study, PRP was prepared manually via centrifugation. The Chinese expert consensus^[34]^ recommends using blood cell separators for PRP preparation, as they offer high integration, a closed environment, controlled platelet concentration, low contamination risk, and greater safety, avoiding technical inconsistencies associated with manual separation and reducing the influence of factors such as centrifugal force, duration, and temperature on PRP quality. PRP prepared by single collection typically has a platelet concentration 4 to 6 times higher than that of whole blood.

Overall, research on PRP for treating thin endometrium after Hysteroscopic Adhesiolysis remains exploratory, with a limited case count. Some scholars are cautious regarding its potential to improve clinical outcomes in patients with moderate-to-severe IUA. Therefore, it is essential to establish standardized, reproducible, and efficient PRP preparation protocols, along with unified treatment plans for PRP timing, dosage, and infusion frequency, to optimize PRP’s role in tissue regeneration. More rigorous clinical trials, larger sample sizes, and extended observation periods are needed to validate its effectiveness.

## Author contributions

**Conceptualization:** Hui-Liu Fan, Hong-Lan Wei, Xiao-Xia Wu.

**Data curation:** Hui-Liu Fan, Hong-Lan Wei, Xiao-Xia Wu, Ying Zhang.

**Formal analysis:** Hui-Liu Fan, Hong-Lan Wei, Xiao-Xia Wu, Ying Zhang.

**Funding acquisition:** Hui-Liu Fan, Xiao-Xia Wu, Ying Zhang.

**Investigation:** Hui-Liu Fan, Hong-Lan Wei, Xiao-Xia Wu, Ying Zhang, Miao-Ling Dou.

**Methodology:** Hui-Liu Fan, Hong-Lan Wei, Xiao-Xia Wu, Ying Zhang, Miao-Ling Dou.

**Project administration:** Hui-Liu Fan, Hong-Lan Wei, Xiao-Xia Wu, Ying Zhang, Miao-Ling Dou.

**Resources:** Hui-Liu Fan, Xiao-Xia Wu.

**Software:** Hui-Liu Fan, Xiao-Xia Wu, Hai-Chun Wei.

**Supervision:** Hui-Liu Fan, Hai-Chun Wei.

**Validation:** Hui-Liu Fan, Hai-Chun Wei.

**Visualization:** Hui-Liu Fan.

**Writing – original draft:** Hui-Liu Fan.

**Writing – review & editing:** Hui-Liu Fan, Hong-Lan Wei.
